# Regular intake of cow’s milk with oral immunotherapy improves statures of children with milk allergies

**DOI:** 10.1016/j.waojou.2020.100108

**Published:** 2020-03-20

**Authors:** Shigehito Emura, Noriyuki Yanagida, Sakura Sato, Ken-ichi Nagakura, Tomoyuki Asaumi, Yu Okada, Yumi Koike, Kiyotake Ogura, Katsuhito Iikura, Motohiro Ebisawa

**Affiliations:** Department of Allergy, Clinical Research Center for Allergy and Rheumatology, National Hospital Organization Sagamihara National Hospital, 18-1 Sakuradai, Minami-ku, Sagamihara-City, Kanagawa, 252-0392, Japan

**Keywords:** Diet, Immunotherapy, Milk allergy, Pediatrics, Cow’s milk, AD, atopic dermatitis, BA, bronchial asthma, Ca, calcium, CM, cow’s milk, CM-Avoid, complete milk avoidance, FA, food allergy, HtSDS, height standard deviation scores, ΔHtSDS, difference in height standard deviation score between the start and end of the observation period, ICS, inhaled corticosteroid, IgE, immunoglobulin E, OFC, oral food challenge, OIT, oral immunotherapy, SD, standard deviation, SDS, standard deviation scores

## Abstract

**Background:**

Children who avoid cow’s milk (CM) because of food allergy may show disturbed growth. Calcium insufficiency, in particular, was reported among those who completely avoided dairy products. We retrospectively examined whether oral immunotherapy (OIT) affected the stature of patients who had completely avoided CM owing to their severe CM allergy.

**Methods:**

The CM-OIT group included subjects who had completely avoided milk their entire lives and were administered OIT between 2009 and 2013. The complete milk avoidance (CM-Avoid) group included subjects who were diagnosed with a CM allergy using oral food challenges between 2013 and 2014 who subsequently avoided CM completely. By examining clinical records and questionnaires, we investigated patient height changes over time. We calculated age- and sex-stratified height standard deviation scores (HtSDS) and analyzed changes in HtSDS retrospectively. The observation period was 1–2 years. To exclude pubertal growth spurts, we set the age criteria as less than 11 years in boys and less than 9 years in girls.

**Results:**

We recruited 29 patients (19 boys) for the CM-OIT group and 20 (9 boys) for the CM-Avoid group. The patients’ median ages at the start of the observation period were 7.5 years (6.1–9.6) for boys and 6.8 years (5.8–7.8) for girls in the CM-OIT group, and 5.4 years (5.0–7.5) for boys and 5.7 years (5.0–7.1) for girls in the CM-Avoid group. The initial HtSDS in the CM-OIT group was −0.31 (median) and increased to −0.22 (median) after OIT (p = 0.016). In contrast, there was no significant change in HtSDS for the CM-Avoid group.

**Conclusions:**

Physical growth of pediatric patients with severe CM allergies, who have avoided CM completely, could be improved by OIT for CM allergy.

## Background

Cow’s milk (CM) allergy is the second most common food allergy next to eggs among children in Japan.^1^ It is known that children can outgrow infantile food allergies, but some children with CM allergy do not develop tolerance compared with patients with hen's egg allergy.^2^ It has been noted that eliminating dairy products completely from one's diet leads to insufficient calcium (Ca) intake,^3^ and it was reported that the amount of CM intake was associated with growth in height.^4^ It was also reported that patients with CM allergy who eliminated dairy products from their diet until they were older than 3 years were shorter than their peers without CM allergy throughout their school years and beyond.^5^ Pediatric patients with CM allergy need to eliminate CM from their diets completely. However, there has been no report evaluating the effects on growth in height after they were again able to consume CM.

In recent years, oral immunotherapy (OIT) has been practiced as a novel approach for food allergy.^6^ At our hospital, we have been administering OIT to several patients with CM allergy, and as a result of successful treatment, CM consumption has increased dramatically in many cases.^7^

Accordingly, this study aimed to clarify the influence of OIT for CM allergy on the stature of children after long-term complete elimination of CM from their diets.

## Methods

### Inclusion criteria and exclusion criteria

The subjects included children who underwent oral food challenges (OFCs) and those tested positive were administered OIT for CM allergy in the department of pediatrics at the hospital between August 2009 and March 2013. The age criteria were between 5 and 9 years old for boys and between 5 and 7 years old for girls at the start of the observation period, and not older than 11 years for boys and 9 years for girls at the end of the observation period, in order to exclude the effect of a rapid growth spurt in height during puberty. In addition, subjects with complications that may affect height, such as congenital esophageal atresia, leptosome, and low birth weight, and those who dropped out of OIT because they were unable to continue to consume CM were excluded from the analysis. Regarding leptosome, we used the definition in the “Health Status Surveillance of School Children” (P16–17), reported by the Public Interest Incorporated Foundation Japan Society of School Health indicating that a subject whose percentage of overweight is −20% or less is thin.^8^

Subjects in the complete milk avoidance (CM-Avoid) group included children who underwent oral food challenges (OFCs) of 3 mL of CM at our hospital between 2012 and 2013. Children who did not meet the above-mentioned age criteria, those with complications that may affect height, and those who were administered OIT within the previous 1 year were excluded from the analysis as well.

By examining the clinical records as well as the questionnaires used for recording the height and weight of the children once measured at the school, kindergarten, or nursery, we investigated patient height changes over the years, number of weeks of gestation, birth weight, CM-specific immunoglobulin E (IgE) value, and the threshold value for the induction of symptoms by CM.

### Evaluation of growth in height

We evaluated height retrospectively based on medical records. We calculated the height standard deviation score (HtSDS) from the mean height and standard deviation by age and sex^9^ and analyzed changes over time during the observation period. The start of the observation period was the date that OIT was first administered to subjects in the CM-OIT group and the date on which the OFC was performed in the CM-Avoid group. The end of the observation period was between 1 and 2 years from the starting date, but typically closer to 1 year. We also analyzed the difference in HtSDS changes from the start of the observation period to the end (ΔHtSDS) between the two groups. ΔHtSDS was calculated using the following formula:

ΔHtSDS = HtSDS at the end of the observation period − HtSDS at the start of the observation period.

Target height was calculated based on a report by Ogata et al.^10^ The equation for boys is target height = {father's height + mother's height +13}/2 (cm), and the equation for girls is target height = {mother's height + father's height - 13}/2 (cm).

In the CM-OIT group, an OFC was conducted using a double-blind placebo control method, with pumpkin cake containing 25 mL of CM. If the subject tested objectively positive, they underwent the OIT. The OIT protocol contained buildup and maintenance phases.^7^ First was the initial build-up phase in the hospital. After that, at home, the patient continued the intake of CM and slowly increased the amount of CM. The target dose of CM was 200 mL. One hundred grams of ordinary liquid CM contains 110 mg of calcium, 3.3 g of protein, 3.8 g of lipid, and 4.8 g of carbohydrate. Throughout the OIT, the patients ingested only CM for the OIT, but they could ingest dairy products or processed foods containing 25 mL of CM if they had been able to ingest 200 mL of CM without immediate reaction for 3 months. In the CM-Avoid group, an OFC was conducted using an open method and pumpkin cake containing 3 mL of CM. If the subject tested objectively positive, they continued to completely eliminate CM from their diets. Both groups had avoided CM completely since birth; and at presentation at our hospital, were directed by the nutritionist to make up for the Ca deficiency by consuming food products containing Ca, rather than milk with hydrolyzed lactoprotein.

### Statistical analysis

The age, HtSDS, number of weeks of gestation, birth weight, CM-specific IgE value, threshold value for the induction of symptoms by CM, and observation period in the patient characteristics data are expressed as the median. The amount of CM intake per day during the OIT period is expressed as the mean. Wilcoxon's signed-rank test was used to compare the HtSDS at the start of the observation period to that at the end of the treatment period and to compare the ΔHtSDS between the groups. The Mann-Whitney *U* test and chi-square test were used to compare patient characteristics between the groups and p < 0.05 was considered significant. Graph Pad Prism (version 6.03, GraphPad Software, La Jolla, Inc., CA, USA) was used for statistical analysis.

## Results

### Subjects

**Fig. 1:**
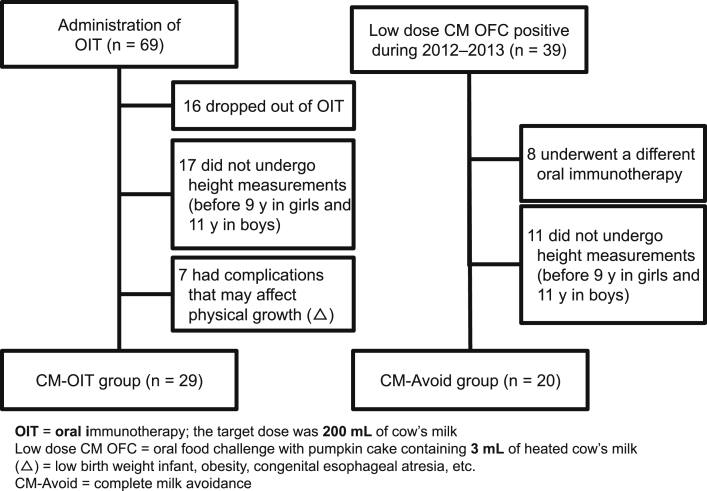
Subject inclusion criteria. OIT, oral immunotherapy; the dose was 200 mL of CM; CM OFC, oral food challenge with pumpkin cake containing 3 mL of heated CM; (†), low birth weight infant, obesity, congenital esophageal atresia, etc.; CM-Avoid, complete milk avoidance

Of the 117 subjects who underwent OIT for CM, 69 subjects met the above age criteria (Fig. 1). Of them, 29 subjects were enrolled in the group to observe the effects of OIT on their height (CM-OIT group). We excluded 17 subjects whose height data was not obtained, 16 subjects who discontinued OIT, and another 7 subjects (2 were low birth weight infants, 1 had congenital esophageal atresia, and 3 were leptosome, and 1 was obese) who had a complication affecting their height.

**Fig. 2:**
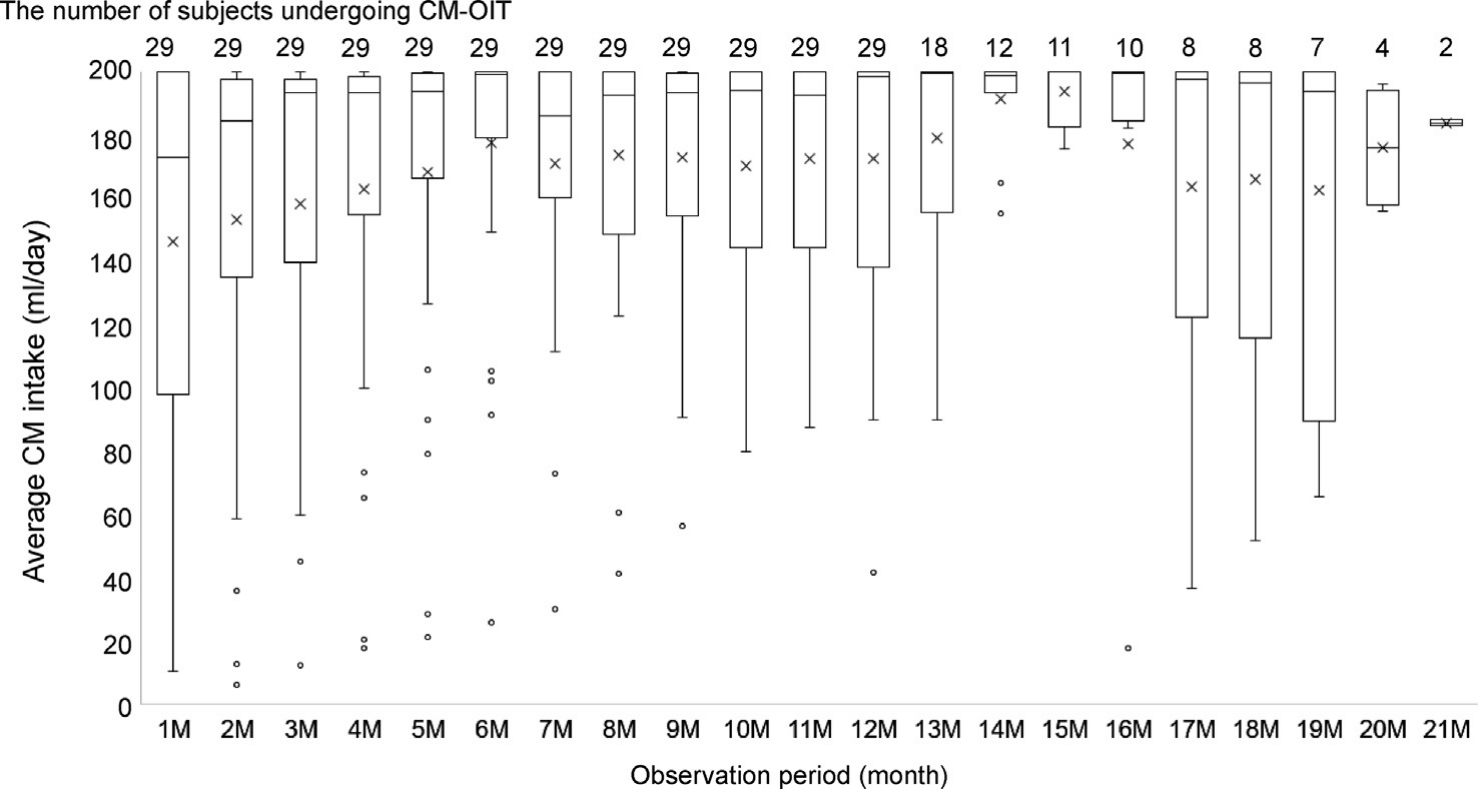
The average CM intake per day in the CM-OIT group during the observation period. CM, cow’s milk; OIT, oral immunotherapy

**Fig. 3:**
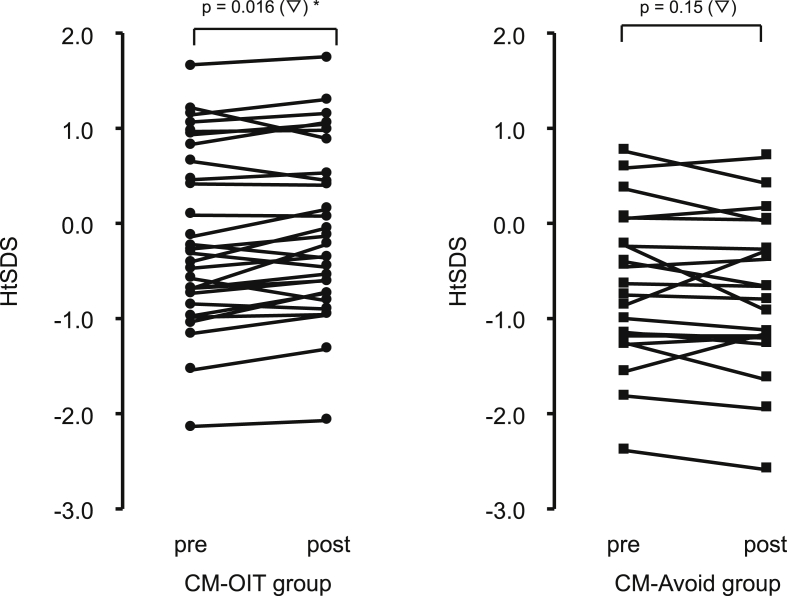
Changes in the standard deviations of the patients' heights. (▽), Wilcoxon's signed-rank test; OIT, oral immunotherapy; HtSDS, height standard deviation score; CM-Avoid, complete milk avoidance

**Fig. 4:**
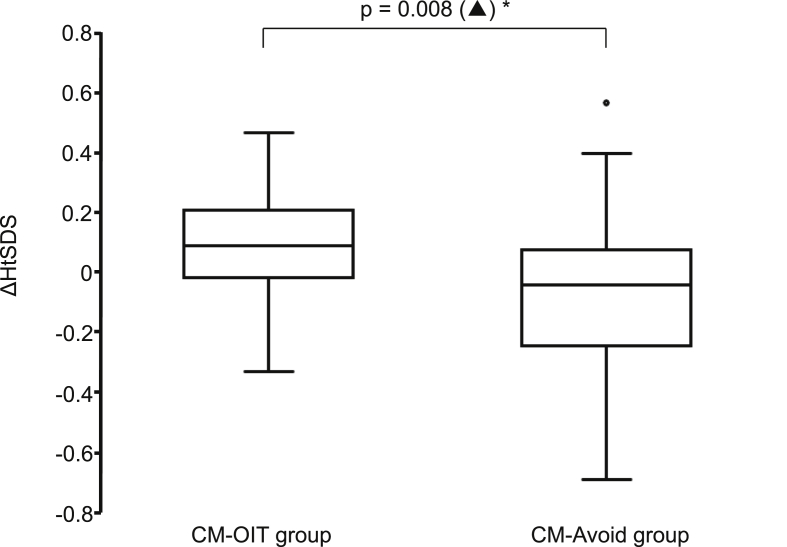
Difference in height standard deviation score between the start and end of the observation period. (▲), Wilcoxon's signed-rank test; OIT, oral immunotherapy; ΔHtSDS, difference in height standard deviation score between the start and end of the observation period; CM-Avoid, complete milk avoidance

Of the 159 pediatric patients who underwent the OFC of pumpkin cake containing 3 mL of heated CM (M0) performed at the hospital from 2012 to 2013, 39 met the age criteria and tested M0-positive and thus were instructed to completely avoid CM. Of these, 20 were enrolled in a group to observe the effects of complete milk avoidance on height (CM-Avoid group), excluding 11 whose height data were not obtained and 8 who had commenced another OIT for CM within 1 year. All patients in the CM-Avoid group had no complications.

### Characteristics of the patients (Table 1)

The CM-OIT group consisted of 19 boys and 10 girls. The CM-Avoid group consisted of 9 boys and 11 girls. The HtSDS at the start of the observation period was −0.31 (−2.14–1.65) in the CM-OIT group and −0.70 (−2.40–0.77) in the CM-Avoid group (p = 0.056). In both groups, there was no case in which the HtSDS at the start of the observation period exceeded the HtSDS of their target height.

**Table 1:** Patient characteristics.

		OIT (n = 29)	CMA (n = 20)	p
sex (male: female)		19:10	9:11	0.15^a^, ^b^
age (years)^b^	male	7.5 (6.1–9.6)	5.4 (5.0–7.5)	0.0001^c^
	female	6.8 (5.8–7.8)	5.7 (5.0–7.1)	0.008^c^
SDS of height^b^		−0.31 (−2.14–1.65)	−0.70 (−2.40–0.77)	0.056^c^
SD of target height		0.38 (−1.53–1.84)	0.47 (−0.83–1.11)	0.71^c^
gestational age (weeks)		40 (37–41)	40 (36–41)	0.37^c^
birth weight (g)		3195 (2606–3840)	3060 (2540–3800)	0.37^c^
FA	avoidance of other foods	3 (1–8)	3 (1–9)	0.15^c^
BA	total, n (%)	18 (62%)	10 (50%)	0.40^a^
	administration of ICS, n (%)	14 (48%)	8 (40%)	0.56^a^
AD, n (%)		26 (89%)	18 (90%)	0.96^a^
cow's milk specific IgE (kUa/L)^b^		58.6 (5.7–412.0)	58.6 (2.7–343.0)	0.67^c^
threshold of cow's milk (mL)^b^		2.1 (0.2–50.0)	3.0 (0.75–3.0)	0.047^c^
observation period (years)		1.1 (1.0–1.8)	1.2 (1.0–1.7)	0.64^c^
intake of cow's milk during OIT (mL/day)^d^		152.8	–	

OIT: oral immunotherapy; SDS: standard deviation score; FA: food allergy; BA: bronchial asthma; ICS: inhaled corticosteroid; AD: atopic dermatitis; CMA = complete milk avoidance.

a Chi square test.

b At the beginning of the observation period.

c Mann-Whitney *U* test.

d Mean

The patients’ ages at the start of the observation period were 7.5 years (6.1–9.6) for boys and 6.8 years (5.8–7.8) for girls in the CM-OIT group, and 5.4 years (5.0–7.5) for boys and 5.7 years (5.0–7.1) for girls in the CM-Avoid group. The symptom induction thresholds for milk were 2.1 mL (0.2–50.0 mL) in the CM-OIT group and 3.0 mL (0.75–3.0 mL) in the CM-Avoid group. There were significant differences between the groups with regard to the age of the patients at the start of the observation period and the symptom induction thresholds for milk.

### Observation period and intake of CM (Fig.2)

The observation periods were 1.1 years (1.0–1.8) in the CM-OIT group and 1.2 years (1.0–1.7) in the CM-Avoid group. The average CM intake per day during the observation period was 176.6 mL in the CM-OIT group and 0 mL in the CM-Avoid group. The CM-OIT group consumed 194.2 mg of calcium per day on average by ingestion of CM.

### Changes in the patients' heights (Fig.3, Fig.4)

In the CM-OIT group, the HtSDS increased significantly from −0.31 (−2.14–1.65) to −0.22 (−2.07–1.74) (p = 0.016). In contrast, in the CM-Avoid group, the HtSDS changed from −0.70 (−2.40–0.70) to −0.74 (−2.60–0.71), and no significant change was found. In addition, the ΔHtSDS in the CM-OIT group was 0.09 (−0.01–0.20) and that in the CM-Avoid group was −0.04 (−0.21–0.07). The ΔHtSDS in the CM-OIT group was significantly higher than that in the CM-Avoid group (p = 0.008).

## Discussion

To the extent we investigated, this study is the first report examining the influence of OIT for CM allergy on growth in height of pediatric patients with severe CM allergy that have completely eliminated CM from their diet.

Among food allergies, it is known that pediatric patients with CM allergies who have completely eliminated CM from their diet show impaired growth in height.^11^, ^12^, ^13^ According to a report by Mukaida et al. on children who eliminated CM from their diet because of food allergy at the age of 3 years, the HtSDS during the school-age period was significantly lower than those of their peers with no food allergy.^5^ Moreover, in our study, no subject was found in which the HtSDS at the start of the observation period exceeded the HtSDS of the target height in the CM-OIT group or CM-Avoid group, and body height growth was observed to be impaired, as was suggested in recent reports.

In this study, the HtSDS in the CM-OIT group increased significantly during the observation period, however, that in the CM-Avoid group did not increase, and there was a significant difference in the ΔHtSDS between the two groups. A desensitization status can be induced in many patients through OIT, even if they were diagnosed with a CM allergy as children and were required to eliminate milk from their diet completely.^7^ This desensitization status refers to a state in which symptoms do not appear in pediatric patients who drink CM daily. Moreover, even when a patient with CM allergy cannot immediately acquire a tolerance to CM, they are able to ingest CM by maintaining a desensitized state.^14^, ^15^, ^16^ Therefore, in the CM-OIT group, the subjects were able to consume a much larger amount of CM than those in the CM-Avoid group. Berkey et al. reported that women who were 9 years old and above with a larger intake of CM demonstrated better growth in height than did women with a lesser intake, and thus, a difference in CM intake can also affect the final height of adults.^4^ The present study has shown a similar result to that suggested in Berkey's report. Moreover, this study suggests that stunted growth in height is not irreversible among pediatric patients with CM allergy who were required to eliminate CM from their diet completely and could be improved by receiving OIT.

A nutritional support service was performed for patients by nutrition specialists regarding supplementary methods for those with insufficient nutrient intake due to CM allergy who eliminated CM from their diet completely.^17^ However, it was reported recently that even though nutritional guidance had been provided to pediatric patients with CM allergy and their parents, their calcium intake remained insufficient.^3^^,^^18^ From these reports, it may be surmised that calcium intake may have been insufficient in the CM-Avoid group. Therefore, it can be estimated that there was a significant difference in calcium intake between the CM-OIT group, where subjects were able to ingest CM in a desensitized state, and the CM-Avoid group, even though the latter had been provided with nutritional advice. Calcium is known to be an essential nutrient for physical development in the growth of children.^19^ Consuming CM during OIT for CM improved growth in height in this study, and it was estimated to be the main factor suggesting that increased calcium intake was due to an increase in CM intake. However, we could not prove this hypothesis that increased calcium intake improved growth in height in the CM-OIT group in this study because we did not measure calcium intake in this study, so future studies are needed to confirm this factor.

There are some limitations to this study. First, there were significant differences in the ages and the symptom induction thresholds between the groups. With regard to the ages, the difference may be derived from the fact that most patients tend to receive OIT for severe food allergy in the summer vacation period after the start of elementary school. Many patients with severe food allergies received OIT at about 6 years of age or older, and the patients in the CM-Avoid group were younger compared with those in the CM-OIT group. Therefore, we evaluated their growth in HtSDS. However, the symptom induction thresholds of the CM-Avoid group were much higher than those of the CM-OIT group. Patients with a severe milk allergy with a low threshold dose tend to receive OIT. However, as both groups had avoided CM completely, there was no difference in CM intake between the two groups before the observation. Second, although there was no significant difference, patients in the CM-OIT group tended to be taller than those in the CM-Avoid group. This may be due to differences in symptom induction threshold and age between the two groups. In the future, it will be necessary to promote treatment in the CM-Avoid group and observe whether the height of the patients in the CM-Avoid group increases or not. Third, the sample size was small because of the study's retrospective nature. Fourth, data on the total calcium intake quantity by CM intake and meals per day is absent in this report. In addition to serum calcium and phosphorus levels, many factors such as the effects of hormones on growth, hormones on calcium metabolism, and bone mineral density were not evaluated. However, increasing the intake of CM among pediatric patients by OIT is considered likely to lead to improvements in height because patients who had complications affecting height were excluded from this study as much as possible, and age conditions were established so that other factors relating to nutrients involved in height development were reduced as much as possible. In the future, a prospective study with a larger sample size would be desirable.

## Conclusion

When certain food elimination diets are required for pediatric patients with food allergy, minimal avoidance should be advised based on a correct diagnosis. In cases that may lead to insufficient calcium intake because of certain food elimination diet, nutritional instructions should be given and controlled, and calcium supplementation by food substitution should be promoted.^17^^,^^20^ However, if the CM allergy has not been reversed and food elimination diets stunt the growth in height of pediatric patients, OIT should be applied as an alternative treatment method whenever possible. In conclusion, this study suggests that for pediatric patients who have avoided CM completely because of a severe CM allergy, physical growth could be improved by introducing OIT for CM.

## Funding

This study was not funded.

## Authors’ contributions

SE performed research, analyzed data, and wrote the paper. KN, TA, YO, YK, KO, and KI collected data. NY supervised SE's work. SS and ME contributed to data analyses and preparation and revision of manuscript. All authors read and approved the final manuscript.

## Ethics approval and consent to participate

The study was carried out in accordance with the ethical principles of the Declaration of Helsinki, it was approved by the ethics committee of Sagamihara National Hospital (2008–7), and written informed consent was taken from all patients and their guardians before they were administered with OIT. Patient anonymity was presented using methods approved by the ethics committee.

## Consent for publication

Not applicable.

## Declaration of Competing Interest

The authors report no competing interests.
